# The Role of Historical Barriers in the Diversification Processes in Open Vegetation Formations during the Miocene/Pliocene Using an Ancient Rodent Lineage as a Model

**DOI:** 10.1371/journal.pone.0061924

**Published:** 2013-04-18

**Authors:** Fabrícia F. Nascimento, Ana Lazar, Albert N. Menezes, Andressa da Matta Durans, Jânio C. Moreira, Jorge Salazar-Bravo, Paulo S. D′Andrea, Cibele R. Bonvicino

**Affiliations:** 1 Laboratório de Biologia e Parasitologia de Mamíferos Silvestres Reservatórios, Instituto Oswaldo Cruz, Fiocruz, Rio de Janeiro, RJ, Brazil; 2 Genetics Division, Instituto Nacional de Câncer, Rio de Janeiro, RJ, Brazil; 3 Programa de Pós Graduação em Biociências, Pavilhão Américo Piquet Carneiro, UERJ, Vila Isabel, Rio de Janeiro, RJ, Brazil; 4 Laboratório de Genômica Funcional e Bioinformática, Instituto Oswaldo Cruz, FIOCRUZ, Rio de Janeiro, RJ, Brazil; 5 Setor de Mastozoologia, Departamento de Vertebrados, Museu Nacional, Universidade Federal do Rio de Janeiro, Quinta da Boa Vista s/n, São Cristovão, Rio de Janeiro, RJ, Brazil; 6 Programa de Pós-Graduação em Biodiversidade e Biologia Evolutiva, UFRJ, Ilha do Fundão, Cidade Universitária, Rio de Janeiro, RJ, Brazil; 7 Department of Biological Sciences, Texas Tech University, Lubbock, Texas, United States of America; University of California, Berkeley, United States of America

## Abstract

The Neotropics harbors a high diversity of species and several hypotheses have been proposed to account for this pattern. However, while species of forested domains are frequently studied, less is known of species from open vegetation formations occupying, altogether, a larger area than the Amazon Forest. Here we evaluate the role of historical barriers and the riverine hypothesis in the speciation patterns of small mammals by analyzing an ancient rodent lineage (*Thrichomys*, Hystricomorpha). Phylogenetic and biogeographic analyses were carried out with mitochondrial and nuclear DNA markers to analyze the evolutionary relationships between *Thrichomys* lineages occurring in dry domains along both banks of the Rio São Francisco. This river is one of the longest of South America whose course and water flow have been modified by inland tectonic activities and climate changes. Molecular data showed a higher number of lineages than previously described. The *T. inermis* species complex with 2n = 26, FN = 48 was observed in both banks of the river showing a paraphyletic arrangement, suggesting that river crossing had occurred, from east to west. A similar pattern was also observed for the *T. apereoides* complex. *Thrichomys* speciation occurred in Late Miocene when the river followed a different course. The current geographic distribution of *Thrichomys* species and their phylogenetic relationships suggested the existence of frequent past connections between both banks in the middle section of the Rio São Francisco. The extensive palaeodune region found in this area has been identified as a centre of endemism of several vertebrate species and is likely to be a center of *Thrichomys* diversification.

## Introduction

The Neotropics harbors a high diversity of species [1] across different biomes, from forest to open vegetation formations. Several hypotheses for explaining its biodiversity, like the refugia and the riverine barrier hypotheses have been tested resulting in contradictory results [2]–[6]. The riverine hypothesis was postulated based on the distribution of primate species with respect to the major Amazonian rivers [7]. This hypothesis predicted that sister taxa would be separated by rivers and that gene flow was more likely to occur in narrow headwater regions rather than downriver sites [8], [9].

Studies of the mammalian fauna across extensive regions of Brazil, a country with both forested and open biomes, will contributed to a better understanding of mammalian speciation timing, in view of its controversial dating to the Tertiary or Quaternary [1], [10]. Furthermore, South American open vegetation domains occupy, altogether, a larger area [11] and may harbor a larger number of mammal species and of endemic species than Amazonia [12], a reason why its biodiversity deserves special attention. Our study focuses on two less frequently studied biomes, the Cerrado and Caatinga.

The Cerrado is the largest open vegetation biome in South America, encompassing an area of approximately 20% of the Brazilian territory and small enclaves in Bolivia and Paraguay [13], [14]. It is the second largest South American biome and one of the most threatened tropical savannas in the world [14], [15]. The Caatinga is one of the largest areas of Seasonally Dry Tropical Forests (SDTFs). It is a poorly studied dry domain encompassing an area of approximately 800,000 Km^2^ and entirely located in Brazil.

Species distribution, biogeography and patterns of historic diversification of open vegetation domains have been recently reviewed by Werneck [11]. This author suggested that the origin and patterns of biodiversity could not be attributed to one or few events during key time intervals. It most likely resulted from complex ecologic and evolutionary trends triggered by Neogene tectonic events and palaeogeographic reorganizations maintained by Quaternary climatic changes and vegetation fluctuations. These areas, infrequently included in phylogeographic studies [16], have become a matter of recent studies [17]–[21] which resulted in earlier estimates of divergence and cryptic diversity.

**Figure 1:**
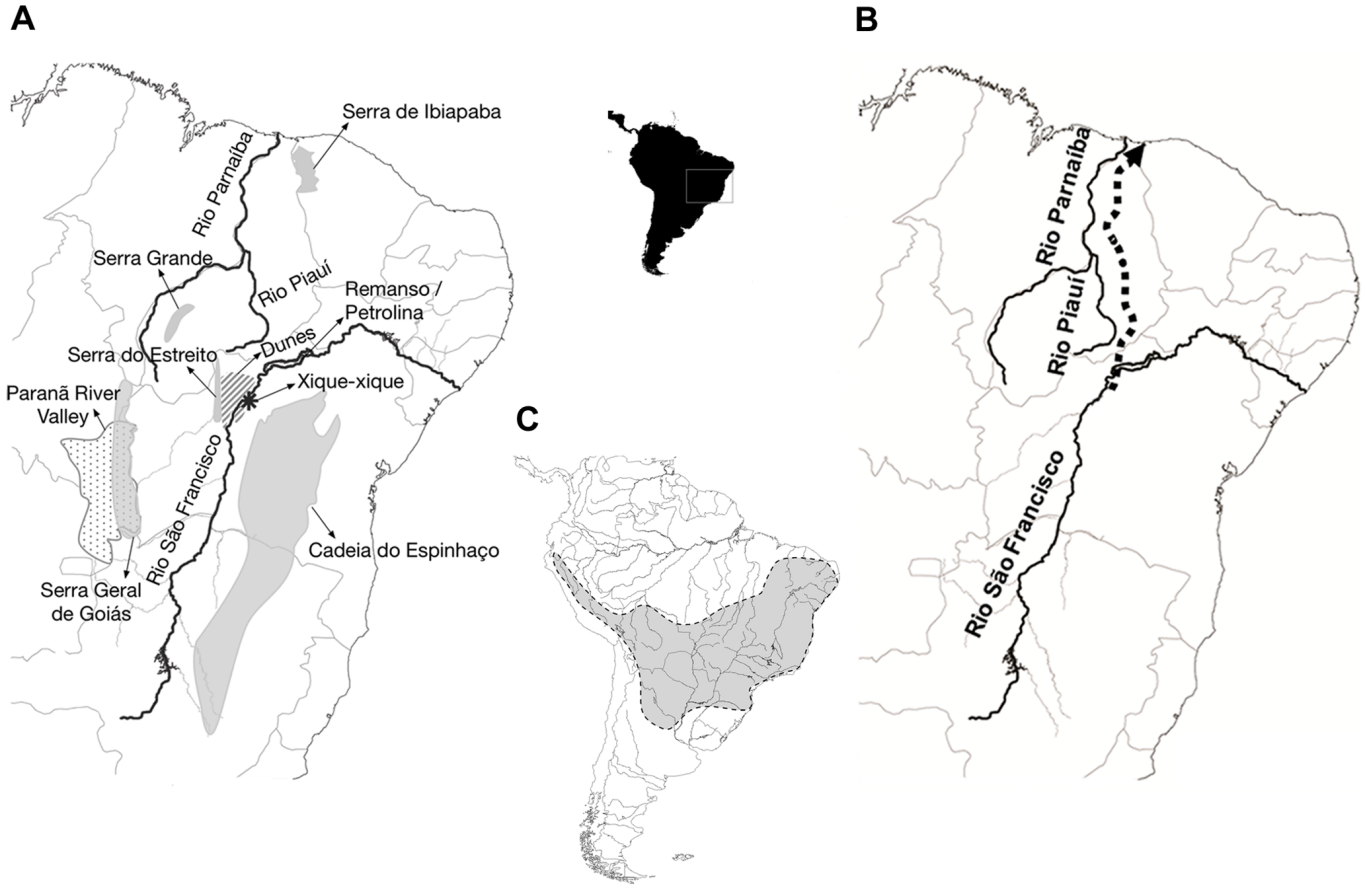
Maps with geological and floristic characteristics showing (A) main rivers and mountain chains; (B) hypothetical course (dotted line) of the Rio São Francisco before switching to its present course; and (C) extent of the "Pleistocenic Arc" following Prado and Gibbs [**97**]. (A) Map showing the main rivers and mountain chains mentioned in the text; (B) Map showing a hypothetical course (dotted line) for the Rio São Francisco before changing to its present course; (C) Map showing the extent of the “Pleistocenic Arc” following Prado and Gibbs [97].

The Rio São Francisco flows through portions of the Cerrado, Caatinga and part of the Atlantic Forest. This river is one of the longest of South America, with the third largest river basin in Brazil, covering an area of approximately 645,000 Km^2^ (nearly 7.6% of the Brazilian territory) [22], [23] within the limits of the São Francisco craton [24]. The maximum width and depth of this river accounts for 850 m and 80 m, respectively, and its annual average flow has been estimated as 2,850 m^3^/s [22]. These characteristics support the proposition that the Rio São Francisco is a barrier to gene flow for several animal taxa. Due to inland tectonic activities, this river is likely to have changed its course [25]–[28] although, presently, it flows towards the north, curving abruptly towards the southeast and to the Atlantic Ocean (Figure 1A). Mabesoone [27] postulated that this river previously flowed in a different direction, probably connecting with the current Rio Piauí and Rio Parnaíba to the equatorial Atlantic Ocean (Figure 1B). This has been supported by the finding of the same gravel deposits of the middle section of the Rio São Francisco and the dry gap between this river and Rio Piauí. Mabesoone [27] also suggested that the course of the Rio São Francisco was interrupted by the uplift of Serra Grande and Ibiapaba cuestas (Figure 1A), subsequently becoming endorheic (stagnated, forming lakes due to lack of outflow) in the Remanso-Petrolina area (Figure 1A), and later changing to its present water course during the Mindel glaciation (*ca*. 450.000 years ago).

During the dry periods of the Late Pleistocene, sand islands emerged due to the lower water level of the Rio São Francisco, favoring the formation of wind-activated dunes [24]. In the Early Holocene, the water volume of the Rio São Francisco was augmented due the increase of humidity and, in the Middle Holocene, the decrease of humidity due to a dry and hot climate favored once again wind activities and expansion of the caatinga vegetation throughout the dunes [24]. At this time, the water volume of the Rio São Francisco probably decreased, once again forming islands similar to the present islands near Xique-Xique municipality [24]. Cyclic climate changes during the Quaternary suggest that the middle course of the Rio São Francisco, close to the sand dunes, is still more prone to forming islands and decreasing its water volume. Furthermore, Barreto *et al*. [29] proposed that the large sand thickness of the dunes area may be attributed to Early Quaternary or even to Late Tertiary events.

To evaluate the riverine hypothesis and how historical barriers have influenced mammalian speciation patterns, we analyzed the evolution of the genus *Thrichomys* based on mitochondrial and nuclear DNA. This rodent genus occurs only in open vegetation biomes and is mainly found in sandy soils and granitic formations including lowland *lajeiros* and elevated *serrotes* and *serras* [30], [31]. *Thrichomys* comprises at least five allopatric species, *T. fosteri*, *T. pachyurus*, *T. inermis*, *T. laurentius* and *T. apereoides*; the last three representing a species complex [32]–[34]. *Thrichomys laurentius*, *T. apereoides* and *T. inermis* are distributed in the Caatinga and Cerrado domains, in both banks of the Rio São Francisco, providing valuable insights on the process of speciation along this river.

## Methods

### Ethics Statement

Small mammals were collected and handled according to recommended safety procedures [35]. Permits for field collection were provided by the Instituto Chico Mendes de Conservação da Biodiversidade (Permit Numbers: 02001.003618/03-06, 02001.006721/2004 and 11375-1). Animal handling procedures were approved by the Animal Research Ethics Committee of the Oswaldo Cruz Foundation, Rio de Janeiro, RJ (CEUA P-0336-07).

Each capture station was sampled with Sherman (7.62×9.53×30.48 cm) or Tomahawk (40.64×12.70×12.70 cm) live traps, placed 10 m apart, in linear transects on the ground. The bait was a mixture of bacon, peanut butter, banana, and oatmeal. Traps were set in the late afternoon and inspected in the early morning.

Each animal was anesthetized and euthanized by intramuscular injection of ketamine hydrochloride (Ketalar; Laboratorio ELEA S.A.C.I.F. y A.) based on an anesthetic protocol according to allometric scaling [36].

### Sample collection and karyotyping

**Figure 2:**
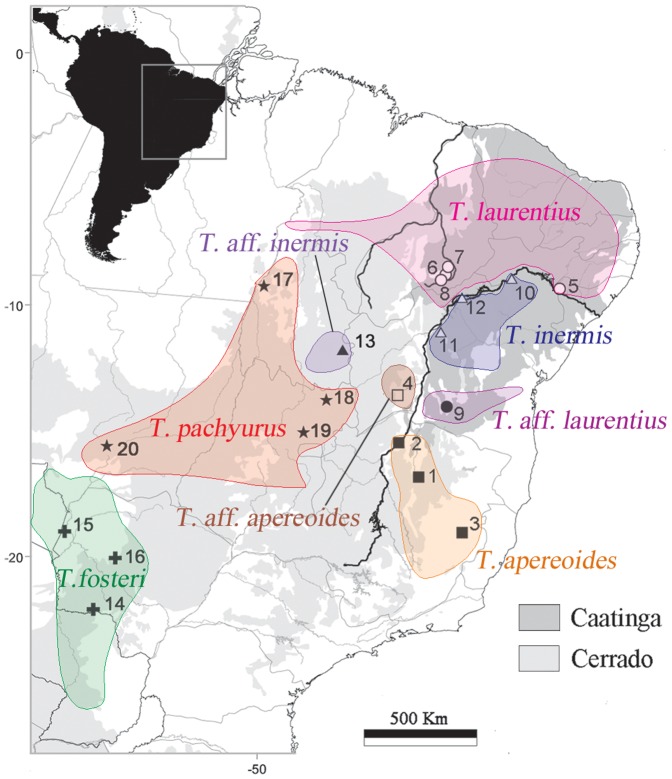
Location of sites of collection of *Thrichomys* (see Table S1 for a list of sites) and outline of *Thrichomys* distribution (modified from [**110**]).

Forty-eight *Thrichomys* were collected in 12 Brazilian municipalities in the Cerrado and Caatinga biomes (Table S1 and Figure 2). We followed the species nomenclature of Braggio and Bonvicino [33], Borodin *et al*. [37] and reassessed the present nomenclature in view of our findings: *T. apereoides* (2n = 28, FNa = 50 located at the right bank of the Rio São Francisco), *T. aff. apereoides* (2n = 28, FNa = 52 at the left bank), *T. laurentius* (2n = 30, FNa = 54 at the left bank), *T. aff. laurentius* (2n = 30, FNa = 54 at the right bank), *T. inermis* (2n = 26, FNa = 48 at the right bank), *T. aff. inermis* (2n = 26, FNa = 48 at the left bank), *T. pachyurus* (2n = 30, FNa = 56) and *T. fosteri* (2n = 34, FNa = 64). *Thrichomys laurentius* was not recognized as a valid species in the latest mammal compilation [38] although a more recent report [37] clearly showed that hybrids between *T. apereoides* and *T. laurentius* were not fertile, supporting species validation. Recently, *T. laurentius* has been recognized as a valid species in the latest Brazilian mammal checklist [39]. In addition, the karyomorphotype 2n = 34, FNa = 64 was considered *T. pachyurus* in the latest mammal compilation. However, specimens collected in Paraguay at Piribebuy, 35 km North of the type locality of *T. fosteri*, showed 2n = 34 and specimens collected by us in the type locality of *T. pachyurus* (Cuiabá, Mato Grosso state) showed 2n = 30, FNa = 56. An outline of *Thrichomys* distribution is shown in Figure 2.

Nineteen specimens were karyotyped in this study (Table S1). Chromosome preparations were obtained from bone marrow cultures in RPMI 1640 medium with 20% fetal calf serum, ethidium bromide [5 μg/mL] and colchicine [10^−6^ M] for two hours. Estimates of fundamental number were restricted to autosome pairs. Voucher specimens are in the mammal collections of the Museu Nacional (MN; Universidade Federal do Rio de Janeiro), and the Laboratório de Biologia e Parasitologia de Mamíferos Reservatórios Silvestres (LBCE; IOC, Fiocruz). Other acronyms refer to field number of C. R. Bonvicino (CRB).

### Gene choice, DNA extraction, PCR assays and sequencing

The haploid mitochondrial genome is uniparently inherited in most animals, is not subjected to recombination and evolves at a faster rate than nuclear DNA. It accounts for a lower effective population size (*N_e_*) when compared to nuclear DNA (nuDNA). These characteristics make mitochondrial DNA (mtDNA) a useful material for evolutionary studies, especially for recently diverged taxa [40]–[42]. On the other hand, noncoding nuDNA harbors a higher variation than coding regions, and is also valuable for analyzing rapidly evolving taxa with a good phylogenetic resolution. Furthermore, the majority of nucleotide positions in introns are free to vary unlike protein coding sequences [43], [44]. Although poorly resolved phylogenies can result by nuDNA analyses due to few informative sites, some studies have shown its utility in phylogenetic reconstructions (for a review see [43]). In this study, we analyzed the mitochondrial cytochrome *b* gene (*cytb*) and the nuclear β-fibrinogen intron 7 (FGB) of *Thrichomys*.

DNA was isolated from 48 liver samples preserved in 100% ethanol with GenomicPrep™ Cells and Tissue DNA Isolation Kit (GE Healthcare, Brazil) or with the standard phenol-chloroform protocol [45].

The *cytb* (1,140 bp) was PCR-amplified using primers L14724 [46] and MVZ14 [47] following a pre-denaturation step at 94°C for 2 min and three series of cycles, of 10, 10 and 15 cycles at 94°C for 30 sec, annealing at 55°C for 30 sec, 53°C for 30 sec, and 51°C for 30 sec respectively, extension at 72°C for 90 sec, and final extension of 72°C for 2 min.

The FGB (*ca*. 800 bp) was amplified in a sample subset (Table S1) with primers β17-mammL and βfib-mammU [48] following a pre-denaturation step at 94°C for 3 min, 5 cycles of denaturation at 94°C for 45 sec, annealing at 62°C (decreasing 0.4°C per cycle) for 45 sec and extension at 72°C for 45 sec; 31 cycles of denaturation at 94°C for 45 sec, annealing at 60°C for 45 sec and extension at 72°C for 45 sec with a final extension of 72°C for 5 min. Due to lack of FGB sequences in GenBank, FGB was amplified in single specimens of *Myocastor coypus* (GenBank JX459849), *Makalata macrura* (JX459850) and *Makalata* sp. (JX459851) to use as outgroups in phylogenetic reconstructions.

Amplicons were purified with GFX™ PCR DNA and Gel Band Purification kit (GE Healthcare). Cytochrome *b* amplicons were labeled with primer L14724 and internal primers TricR (5′-TGTGATGACTGTCGCTCCTC-3′), cytbTrich (5′-CCCTACATCGGACCTTCACT-3′) and MVZ16 [47], while FGB amplicons were labeled with primers β17-mammL and βfib-mammU. Sequencing was carried out with ABI Prism^TM^ 377 and MegaBACE 1000 (GE Healthcare) platforms. Electropherograms were manually checked using Bioedit (version 7.0.8.0) [49].

### Alignment and genetic distance estimation

A total of 70 *Thrichomys cytb* were analyzed. These included the complete (1,140 bp) *cytb* sequence of three *T. laurentius*, two from Coronel José Dias municipality (AY083333, AY083334) and another from João Costa municipality (AY083332) previously analyzed by Braggio and Bonvicino [33] based on 817, 1,109 and 801 base pairs, respectively. Twenty-two of these 70 sequences have been previously published [33], [50], [51] (Table S1). Furthermore, one sequence of *Ctenomys boliviensis* (AF155869), *Ctenomys maulinus* (AF370703), *Euryzygomatomys spinosus* (U34858), *Dasyprocta leporina* (AF437789), *Hydrochoeris hydrochaeris* (GU136721), *Erethizon dorsatum* (FJ357428), *Proechimys cuvieri* (AY206631), *Makalata macrura* (EU302702) and *Myocastor coypus* (EU544663) were used as outgroups. A total of 18 FGB sequences (*ca*. 800 bp), representative of each *Thrichomys* lineages, were analyzed. One sequence of *Myocastor coypus* (JX459849) and *Makalata macrura* (JX459850) were also included in phylogenetic analyses. All sequences were manually aligned with Bioedit.

Pairwise genetic distances were estimated using the modified Log-Det for closely related taxa [52] using Mega 5 [53].

### Gene tree estimation

A DNA substitution model was selected prior to phylogenetic reconstructions using Modelgenerator version 0.85 [54] and the Bayesian information criterion (BIC).

Gene tree estimation was based on *cytb* and FGB carried out with maximum likelihood (ML) using PhyML version 3.0 [55] and Bayesian inference (BI) using MrBayes 3.2 [56].

The ML tree topology was searched with the best of Nearest Neighbor Interchange and Subtree Pruning and Regrafting algorithms from five random, starting trees generated by the BioNJ algorithm [55], [57]. Branch support was estimated using the approximate likelihood ratio test with Shimodaira-Hasegawa-like interpretation (SH-aLRT), a procedure as conservative and accurate as bootstrapping but less computationally intensive [57]–[59]. For BI, analyses were run for 4,000,000 generations with sampling every 100. Acceptable mixing and convergence to the stationary distribution were checked with Tracer 1.5 [60] and the first 10% were discarded as burn-in. A consensus tree was then generated. We used five complete *cytb* sequences of *T. laurentius* (one representative of each location).

### Species tree and divergence date estimation

To estimate the *Thrichomys* species tree we used the *Beast (StarBeast) method [61] implemented in Beast 1.7.0 [62] using both *cytb* and FGB genes and unlinked substitution models, clock models and trees. An uncorrelated lognormal relaxed clock [63] and a Yule tree prior [64] were used and estimates of posterior distribution were obtained by Markov chain Monte Carlo (MCMC) sampling every 5,000 MCMC steps over a total of 100,000,000 steps. As a higher number of *cytb* sequences were generated, and to avoiding a bias resulting from missing data, the species tree was reconstructed based on specimens for which *cytb* and FGB data were available.

We calibrated *Thrichomys* divergence based on its sister fossil taxon *Pampamys emmonsae*, Echimyidae from Cerro Azul Formation, La Pampa Province, Argentina, of Chasicoan-Huayquerian age [65]–[67]. This accounted for a minimum age constraint of 6.0 Ma as suggested by Olivares *et al*. [65] and a maximum age constraint of 10 Ma based on the boundary between the Chasicoan and Huayquerian ages. Estimates of divergence times were calibrated using a lognormally distributed prior for the divergence of *Thrichomys*. Lognormal prior was preferred in view of its most appropriate distribution to summarize paleontologic information [68].

We included a user generated start tree based on a nonparametric rate smoothing (NPRS) transformed consensus tree estimated with MrBayes. Acceptable mixing and convergence to the stationary distribution were checked using Tracer and the first 10% were discarded as burn-in. The maximum clade credibility (MCC) tree was computed with TreeAnnotator and using mean heights for divergence date estimates.

### Phylogeographic analysis

In order to reconstruct the phylogeographic history of *Thrichomys* species through time and in continuous space (by using location coordinate data for specimens), we used the relaxed random walk (RRW) approach proposed by Lemey *et al*. [69]. We used the Cauchy RRW model implemented in Beast and all other parameters were set as previously described in the species tree and divergence date estimation section. Because some specimen coordinates were duplicated we used a jitter option of 0.50. Estimates of the posterior distribution were obtained by MCMC sampling every 5,000 MCMC steps over a total of 50,000,000 steps. This analysis was performed for *cytb* excluding identical haplotypes and outgroups.

We also included a user generated start tree based on a nonparametric rate smoothing (NPRS) transformed consensus tree estimated with MrBayes. Acceptable mixing and convergence to the stationary distribution were also checked using Tracer and the first 10% were discarded as burn-in. The MCC tree was computed with TreeAnnotator and using mean heights for divergence date estimates.

To summarize the posterior distribution of ancestral locations using the Cauchy RRW model we annotated nodes in a MCC tree using the program TreeAnnotator. This MCC tree was used as input for the program Spread 1.0.4 [70] to generate a keyhole markup language (KML) file containing the reconstructed dispersal route paths to visualize in Google Earth. Although this reconstructed route of dispersal has been superimposed on a contemporary map rather than on an unavailable Tertiary map, it provides useful information on locations of ancestral *Thrichomys* populations and their distribution along SDTFs.

### Biogeographic reconstructions

In order to reconstruct the biogeographic history of *Thrichomys* we employed a parsimony-based reconstruction method using a modified version of the dispersal-vicariance analysis (DIVA) [71]–the S-DIVA (Statistical DIVA). Analyses were carried out with *cytb* data with Rasp [72] and implementing the “Bayes-DIVA” approach [73]. We used the complete tree distribution obtained with the phylogeographic Beast analysis without the first 10% as burn-in to account for phylogenetic uncertainty and the MCC tree. The maximum area at each node was set to 2 [74]. Biogeographic regions were defined *a priori* (Table S1 and Figure S3).

### Migration rates

To further test whether the Rio São Francisco might be a potential barrier to gene flow we estimated migration rates (M) between populations using both IMa2 [75], [76] and Migrate [77], [78] with the *cytb* dataset. Because we analyzed populations of different *Thrichomys* species, we compared population pairs: (1) between river banks and sharing the same karyotype (*T. apereoides versus T. aff. apereoides*, and *T. inermis versus T. aff. inermis*), (2) from the same river bank and geographically close (*T. pachyurus versus T. fosteri*, *T. pachyurus versus T. aff. inermis*, and *T. inermis versus T. apereoides*), (3) from the same river bank but geographically distant (*T. laurentius versus T. fosteri*). If the river were a barrier to gene flow, then lower migration rates would be expected between population pairs from opposite banks than between population pairs from the same bank.

After preliminary runs to check convergence of parameters, IMa2 was run in the L-mode with different start seeds and an initial burn-in of 100,000 steps followed by saving 500,000 genealogies every 100th MCMC steps. Migrate was run in the Bayesian inference mode with an initial burn-in of 50,000 steps for 500,000 (or 2,000,000) MCMC steps. We used the static heating with four chains sampled at every tenth interval using the temperature scheme suggested with the character # as described in the Migrate tutorial [79].

## Results

### Karyologic analyses

*Thrichomys aff. apereoides* from the left bank of the Rio São Francisco showed 2n = 28 and FNa_ = _52, while *T. apereoides* from the right bank of this river showed 2n_ = _28 and FNa_ = _50. *Thrichomys inermis* from the right bank of the Rio São Francisco and *T. aff. inermis* from the left bank of this river showed the same 2n_ = _26 and FNa_ = _48 karyotype. *Thrichomys pachyurus* from type locality showed 2n = 30 and FNa_ = _56. *Thrichomys laurentius* and *T. aff. laurentius* also showed the same 2n_ = _30 and FNa_ = _54 karyotype. Finally, *T. fosteri* showed 2n_ = _34 and FNa = 64 (Table S1).

### Descriptive analyses of *cytb* and FGB intron

Table S1 lists sequence data from all *Thrichomys* specimens belonging to eight lineages herein analyzed. Analysis of *cytb* and FGB showed 59 and 10 haplotypes, respectively, none of them being shared between different taxa. We also observed a higher proportion of variable and parsimony-informative sites for *cytb* (24.3% and 18.7%, respectively) than FGB (4.1% and 2.9%, respectively).

Pairwise genetic distances for *cytb* within *T. apereoides* (from right bank of the Rio São Francisco) were higher (0.0–1.9%) than within *T. aff. apereoides* (from left bank) (0.0–0.3%), while genetic distance estimates between these lineages ranged from 2.8–3.9%. Pairwise genetic distances within *T. laurentius* (from left bank) ranged from 0.0–3.1% unlike *T. aff. laurentius* (from right bank) which showed a single haplotype. Genetic distance between these lineages ranged from 3.7–4.6%. Pairwise genetic distances within *T. inermis* (from right bank) were higher (0.0–9.6%) than within *T. aff. inermis* (from left bank) (0.0–1.3%) while estimates between these lineages equaled 7.5–10.4%. Genetic distance within *T. fosteri* and *T. pachyurus* equaled 0.0–1.5% and 0.0–2.9% respectively.

The FGB in *T. apereoides* specimens from the right bank showed a single haplotype that differed from the one found in the single specimen of *T. aff. apereoides* from the left bank. Genetic distance between these two lineages was 0.6%. FGB in *T. laurentius* from the left bank showed a single haplotype that differed from the single haplotype of *T. aff. laurentius* from the right bank. Genetic distances between these two lineages were 1.1%. Pairwise genetic distances in *T. inermis* from right bank were higher (0.0–1.6%) than in *T. aff. inermis* from left bank (0.0–0.1%), while estimates between these lineages ranged from 1.1–1.3%. Single haplotypes were observed in *T. fosteri* and *T. pachyurus*.

### Gene trees

Phylogenetic trees based on *cytb* were constructed with HKY [80] and gamma distribution as DNA substitution model. Both ML and Bayesian trees (Figure S1) showed very similar topologies; all branches were well supported except for *T. aff. inermis* (branch support≈0.60; Figure S1). All analyses showed a paraphyletic arrangement for *T. inermis* between opposite banks of the Rio São Francisco despite sharing the same karyotype with conventional staining. In view of these findings, *T. inermis* from the left bank was renamed *T. aff. inermis*. A close relationship was also observed between *T. aff. apereoides* and *T. aff. laurentius*, currently separated by the Rio São Francisco. All analyses were also consistent in showing *T. inermis*, currently located in the right bank, as the most basal lineage.

Phylogenetic trees based on FGB (Figure S2), constructed with K80 [81] and gamma distribution as DNA substitution model yielded less robust resolution of the evolutionary relationships of *Thrichomys*. Branch support for the node between *T. fosteri* and the remaining lineages of the clade was low (0.67; Figure S2) as was the case of the node between *T. pachyurus* and the remaining lineages of the clade (0.52; Figure S2). These low estimates might have resulted from a lower number of variable and parsimony-informative sites in FGB than in *cytb*. Despite these limitations, however, FGB topologies were consistent in showing *T. inermis* as the most basal lineage.

### Species tree, phylogeographic analysis and divergence dating

**Figure 3:**
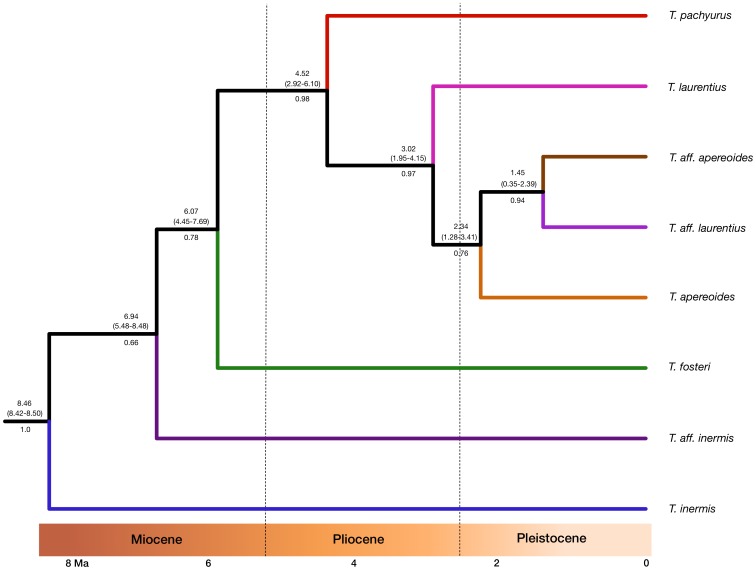
MCC phylogeny representing *Thrichomys* species tree generated with the *Beast method. Numbers above branches are mean height and 95% HPD interval (in brackets) of date estimates and numbers below branches are posterior probability values. This analysis was generated using Beast. Branch colors follow same colors of Figure 2.

The *Thrichomys* species tree (Figure 3) obtained with *Beast showed an identical topology to the *cytb* gene tree (Figure S1). All branches were well supported except for those leading to *T. aff. inermis*, *T. fosteri* and *T. apereoides*.

Divergence time estimates showed that the ancestral *Thrichomys* population had been present in the Late Miocene [8.46 Ma (highest posterior density (HPD) = 8.42 to 8.50 Ma)] and that most lineages emerged in the Plio-Pleistocene (Figure 3). Interestingly, the ancestors of each *Thrichomys* lineage apparently originated at similar times, as indicated by overlapping between 95% HPD estimates (Figure 3). *Thrichomys apereoides*, *T. aff. apereoides*, *T. laurentius* and *T. aff. laurentius* comprise a clade that diverged in the Pliocene [3.02 Ma (1.95 to 4.15)] (Figure 3).

**Figure 4:**
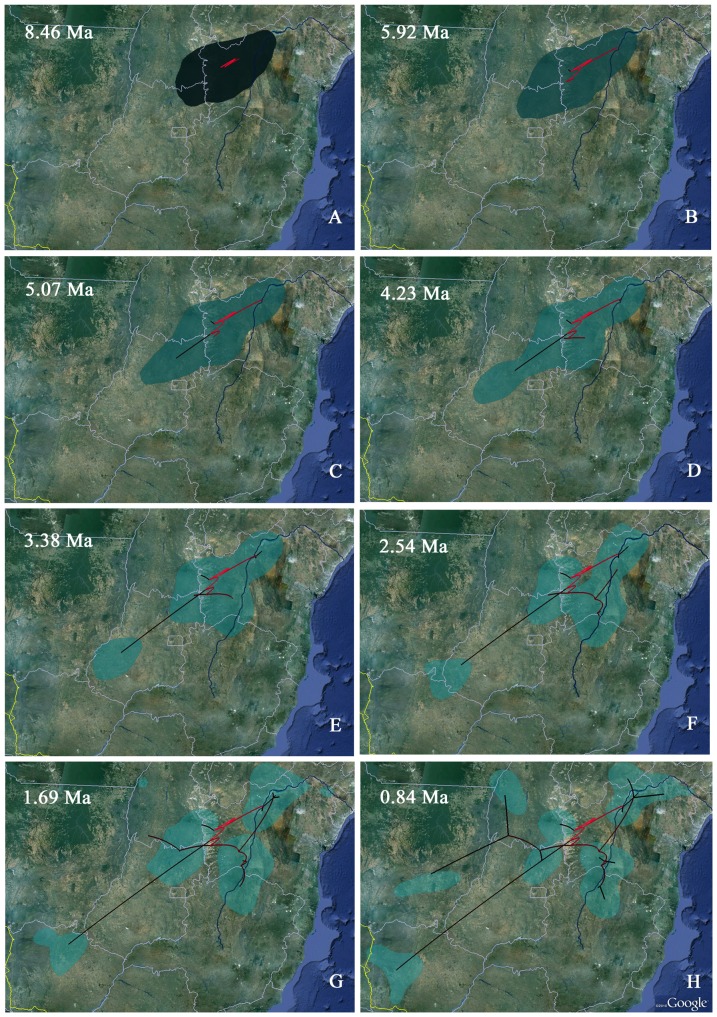
Spatiotemporal dynamics of *Thrichomys* suggesting they originated in both banks of the Rio São Francisco from 8.46 to 5.92 Ma (A–B), a posterior range expansion occurred at *ca*. 5.07 to 4.23 Ma ago (**C–D**), a population split in two demes at *ca*. 3.38 Ma (**E**), another expansion of an ancestral population at *ca*. 2.54 Ma (**F**), and the emergence of six ancestral populations at *ca*. 1.69 Ma (**G**) that coexisted for approximately 0.84 Ma (**H**). Lines represent the MCC tree branches projected on the surface. Maps are based on satellite pictures made available with Google Earth. The current course of the Rio São Francisco is highlighted.

Phylogeographic analysis suggested that *Thrichomys* originated in central Caatinga and northern Cerrado, in both banks of the Rio São Francisco (Figure 4A). A similar area to the one presently existing remained unchanged for a long period, probably until 5.92 Ma (Figure 4B), while, approximately 5.07 to 4.23 Ma ago, a range expansion probably occurred towards the southwest (Figure 4C and 4D). The ancestral *Thrichomys* population split in two demes at approximately 3.38 Ma (Figure 4E). Approximately 2.54 Ma, the ancestral population expanded (Figure 4F) and, approximately 1.69 Ma, six ancestral populations emerged, one of them accounting for the current distribution of *T. fosteri* (Figure 4G).

Six ancestral populations coexisted approximately 0.84 Ma, one of them still accounting for the current distribution of *T. fosteri*, three other to the current distribution of *T. pachyurus* and *T. aff. inermis*, a fifth one to the current distribution of *T. laurentius* and *T. inermis*, and a sixth one to the current distribution of *T. aff. laurentius*, *T. apereoides* and *T. aff. apereoides* (Figure 4H).

### Biogeographic reconstructions

**Figure 5:**
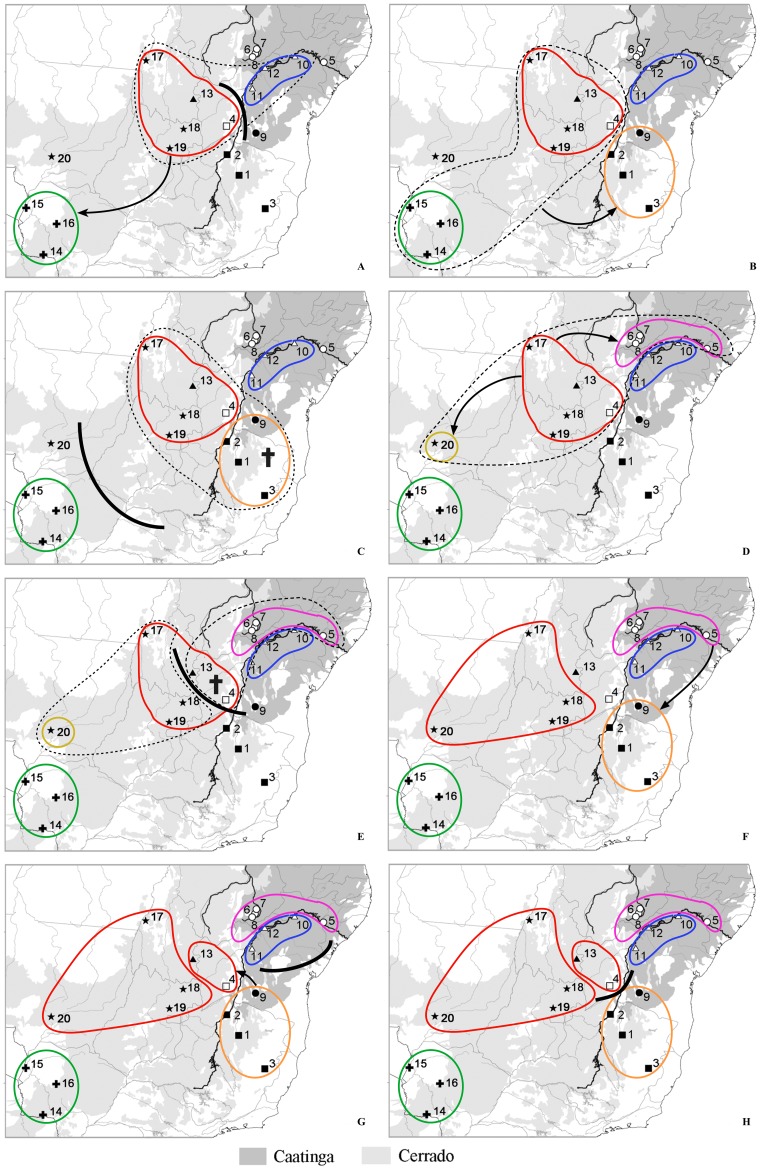
Biogeographic hypotheses of colonization history of *Thichomys* based on S-DIVA analysis using RASP (A–H). Solid line polygons represent the different ancestral populations; dotted line polygons represent past connected populations; solid lines represent vicariant events; crosses represent extinctions; arrows represent dispersions. Sites of collection are the same as Figure 2 and Table S1 and they are represented here for clarity.

S-DIVA analyses indicated that the ancestral *Thrichomys* population originated with a probability of 65% in the central Caatinga and/or the northern Cerrado, probably in both banks of the Rio São Francisco. It also indicated that a vicariant event separated this ancestral population but soon after the population of the northern Cerrado dispersed to the southern Cerrado (Figure 5A). These two populations dispersed to the southern Caatinga across the river (Figure 5B), followed by a vicariant event separating the population of the southern Cerrado from the northern Cerrado and southern Caatinga, accounting for the current distribution of *T. fosteri* (Figure 5C).

Populations of the southern Caatinga and the one from northern Cerrado became extinct in the southern Caatinga (Figure 5C) and, subsequently, the remaining ancestral population of the northern Cerrado dispersed to the central Cerrado and northern Caatinga (Figure 5D). Soon after, a vicariant event split the ancestral populations of the northern Cerrado forming two separate populations: one with the population of the central Cerrado and another with the population of the northern Caatinga (Figure 5E).

Part of the ancestral population of the northern Cerrado which dispersed to the northern Caatinga became extinct (Figure 5E). The population of the northern Caatinga probably originated the current *T. laurentius* and dispersed to the southern Caatinga (Figure 5F), accounting for a second colonization event in this area. Soon after, a vicariant event separated populations of the northern from the southern Caatinga (Figure 5G), an event likely related to the initial alterations of the river course.

The ancestral population of the southern Caatinga dispersed to the northern Cerrado (Figure 5G) followed by a vicariant event separating both populations (Figure 5H).

### Migration rates

Migrate detected low migration between population pairs (Table 1) while IMa2 did not detect migrations, suggesting that migration did not occur between any population pairs.

**Table 1:** List of populations tested with estimates of migration rates from Migrate.

Pairs of populations tested	aM_2→1_	aM_1→2_
*T. apereoides* and *T. aff. apereoides*	95.0 (0.0–830.7)	145.0 (0.0–870.0)
*T. inermis* and *T. aff. inermis*	52.3 (0.0–848.0)	467.0 (3.3–900.7)
*T. fosteri* and *T. pachyurus*	37.0 (0.0–771.3)	15.7 (0.0–843.3)
*T. pachyurus* and *T. aff. inermis*	15.0 (0.0–777.3)	31.0 (0.0–847.3)
*T. inermis* and *T. apereoides*	189.0 (8.7–863.3)	461.0 (136.0–984.7)
*T. laurentius* and *T. fosteri*	21.7 (0.0–866.7)	29.0 (0.0–777.3)

a Modes are reported, as curves were positively skewed. Numbers in brackets represent 95% confidence intervals. Subscripts 1 and 2 represent the order in which species were listed in the first column.

## Discussion

### Mitochondrial and nuclear gene tree discordances and the gene tree *versus* species tree

Our results showed a disagreement between *cytb* and FGB gene trees, a common result in phylogenetic and phylogeographic studies (for a review see [82]). One reason for this pattern was attributed to long-term isolation followed by current secondary population contact or population range contact at some point in the past [82]. Our analyses showed *Thrichomys* as a very ancient lineage that possibly exchanged migrants in the past because of fluctuation in the river water volume allowing crossings between river banks (see below).

Furthermore, discordances between mitochondrial *versus* nuclear gene trees can be a consequence of the effective population size (*N_e_*), which in most circumstances is smaller for mtDNA [83]. In general, smaller *N_e_* increases the probability of congruence between gene and species trees and, in most cases, mtDNA will be more likely congruent to the species tree [84]. However, for some extreme scenarios a nuDNA would be favored over mtDNA for obtaining the species tree [84], [85]. Our results showed a species tree (Figure 3) identical to that using the *cytb* gene (Figure S1) suggesting that for *Thrichomys*, mtDNA would be a reliable choice for recovering the species tree. However, other nuclear genes should be analyzed for a better resolution of this assumption.

### Climate change, diversification processes and the Rio São Francisco

Our results suggest that the ancestral *Thrichomys* population initially appeared in the Late Miocene [8.46 Ma (8.42 to 8.50 Ma)]. This very ancient origin presumably explained why Migrate and IMa2 were incapable of detecting migration between population pairs. Lack of gene flow also indicated that all eight *Thrichomys* evolutionary lineages (including the *affinis* species) are well defined and reproductively isolated species.

Our estimates of *Thrichomys* divergence differed from previous reports postulating an earlier origin [86]–[88]. Leite and Patton [86] used the same fossil record (*Pampamys emmonsae*) as the one used in this report albeit dated to the Huayquerian age (6.8 to 9.0 Ma) following Verzi *et al*. [67]. Galewski's *et al*. [87] single constraint for Caviomorph radiation was based on the fossil record of the Dasyproctidae and Chinchilidae from the Tinguiririca fauna of Chile dated between 31.5 to 37.5 Ma [89], [90]. Although their dating of *Thrichomys*/*Proechimys* divergence was estimated as 13.3 Ma, their 95% credibility interval was very high (7.4 to 20.7 Ma), probably due to relying on a single and ancient calibration point at the root of their phylogenetic tree. Upham and Patterson [88], based on five calibration points, dated the splitting of *Thrichomys* from *Hoplomys*/*Proechimys* to approximately 12 to 16 Ma. However, this node was not calibrated, a reason why the origin of *Thrichomys* could not be precisely estimated. Conversely, in our report, the node of the *Thrichomys* clade was calibrated based on the fossil record of its sister taxon *P. emmonsae* (see Candela and Rasia [91]) and the whole suite of congeneric species was analyzed.

Our results also suggest that the described scenario of water volume oscillations of the Rio São Francisco also occurred in other geological periods, like the Miocene. These oscillations lead to a decrease of the water volume and island formations around 10°–12° S, close to the sand dunes of the middle section of this river, suggesting ancient connections between river banks in the region close to the dunes and influencing the distribution and diversification of *Thrichomys*. This diversification occurred after the geologic origin of the Rio São Francisco, dated to the Early-to-Mid Cretaceous [92]. Furthermore, the species tree (Figure 3) suggests that the riverine hypothesis does not hold for *Thrichomys* species, with the exception of *T. aff. apereoides* and *T. aff. laurentius*. These two lineages are more related to each other and are currently separated by the river, following the riverine hypothesis.

The paraphyletic arrangement of *T. inermis* complex observed in phylogenetic analyses suggested two different evolutionary lineages. This taxon crossed the middle section of the Rio São Francisco, probably from east to west in view that *T. inermis* from Bahia (in the right bank of the river) appeared as the most basal and oldest lineage. This could also explain why a similar karyotype was found in populations of both banks of the river and why these populations are more closely related, implying a past continuous contact between them as indicated by phylogeographic analysis, at least before 0.84 Ma (Figure 4). *Thrichomys aff. inermis*, currently located in left bank of the Rio São Francisco, was collected in Novo Jardim, in areas of SDTF enclaves of the Cerrado, located in the Paranã River Valley (Figure 1A). This valley encompasses an area of 5,940,382 ha in southeast Tocantins and northeast Goiás, in a transition area of the Cerrado, Caatinga and Amazon Forest [93], [94], and delimited in the east by the Serra Geral de Goiás (Figure 1A), a mountain chain between the Cerrado and Caatinga [95]. This region shows a high diversity of phytophysiognomies and is one of the most heterogeneous SDTF enclaves of Brazil [93]. These SDTFs currently show a disjunct distribution, a likely relict of a more extensive and continuous region hypothesized as the “Pleistocenic Arc” [11], [96], [97] (Figure 1C). This arc, evident by the distribution of tree species [97], might have ranged from northeastern to southeastern Brazil, northwestern Argentina, southwestern Bolivia and northwards to the dry Andean valleys of Peru, indicated that populations showed a more continuous and wide distribution in the past. Recently, it has been shown that, during the Late Pleistocene, dry regions were more disjunct than present ones, suggesting their previous expansion in the Early Pleistocene or end of the Tertiary, subsequently fragmented in the last glacial period and later undergoing a further expansion in the Holocene [11]. In this scenario, *Thrichomys* populations from the Caatinga might have colonized regions of the Cerrado (Figure 5), occupying present SDTF enclaves, a pattern suggested by our biogeographic analysis in view that most *Thrichomys* lineages emerged in the Plio-Pleistocene. Subsequently, these populations became isolated, followed different evolutionary histories and suffered differentiation, establishing endemic centers [11], [15], [98]. Plant, lizard, bird and mammal species endemic to STDF enclaves have been reported [13], [15], [98]–[100]. Furthermore, lizard species endemic to Caatinga were found in these enclaves, being completely isolated from other populations of Caatinga [98], suggesting a dynamic historic connection between the Cerrado and Caatinga, a pattern similar to the one observed for *T. inermis* and *T. aff. inermis* populations, both with 2n = 26, FNa = 48. These findings suggested that a vicariant event separated the ancestor of these *Thrichomys* lineages (Figure 5). This ancestor probably occurred along the “Pleistocenic Arc” and later split when STDFs became discontinuous, originating two isolated populations.

Further evidence on the coincidence between climate change and dispersal across the Rio São Francisco was provided by the close relationship of *T. aff. apereoides* and *T. aff. laurentius*, both from Bahia state and currently separated by this river, as well as the close relation of *T. laurentius* from the left bank with other species from the right bank (*T. aff. laurentius* and *T. apereoides*). This pattern corroborated Mabesoone's [27] hypothesis that the course of the Rio São Francisco differed from the present one due to its opening to the equatorial Atlantic Ocean rather than to the northeast. A ancient river course similar to the present one would result in a closer relationship of *T. laurentius* with species of the left bank (*T. aff. inermis*, *T. pachyurus* and *T. fosteri*) rather than to species of the right bank.

The Rio São Francisco is also a natural geographic barrier influencing the genetic diversification and speciation of different taxa, like lizard species [101]–[103], rodents [104] and sand flies [105]. The middle course of the Rio São Francisco and its paleodunes appear to be a dry refuge for open vegetation inhabitants. We postulate that this dry refuge is a center of endemism in this region, well documented by one mammal, one bird species, 20 new species and four new genera of squamates [106]–[108]. This dry refuge apparently contributed to speciation patterns of *Thrichomys*, with the oldest species, *T. inermis*, occurring near the paleodune area.

### The dry morphoclimatic domains and *Thrichomys* diversity

The open vegetation formations of the Caatinga and Cerrado domains were probably reduced to isolated patches associated to speciation of some faunal taxa during the Quaternary-Tertiary, affecting the distribution of *T. apereoides*, *T. inermis* and *T. laurentius* species complexes. Most localities of *T. apereoides* (2n = 28, FNa = 50), including its type locality, are found in the right bank of the Rio São Francisco [32], [33], [109], mainly in the Caatinga domain, but also in the Cerrado, while *T. aff. apereoides* (2n = 28, FNa = 52) occurs in the left bank of this river in the Cerrado [32]. Similarly, all *T. inermis* localities, including its type locality, are in the right bank of the Rio São Francisco, restricted to the Caatinga, while the left bank *T. aff. inermis*, is restricted to the Cerrado. However, *T. laurentius* complex, although separated in two evolutionary lineages, does not follow this clear pattern. All localities of *T. laurentius*, including its type locality, are found in the left bank of this river contrary to all localities of *T. aff. laurentius* in the right bank [33], both in the Caatinga domain. The relief around the middle course of the Rio São Francisco is complex with several mountain chains, like the Cadeia do Espinhaço, Serra Geral de Goiás and Serra do Estreito (Figure 1A) which are apparent barriers to *Thrichomys*.

## Conclusion

The diversification in the studied area cannot be explained by the riverine hypothesis. Due to the older age of the Rio São Francisco compared to *Thrichomys* origin, it is more likely that the pattern observed was a consequence of climate changes and arid periods in the middle section of the river. The cyclic increase and decrease of water volume appear to have allowed for interruptions of gene flow or, alternatively, crossings across riverbanks, influencing speciation and biological diversity. Our results also suggest a role of the ancient course of the river in the diversification of *Thrichomys*. Contrary to forested domains where a high biodiversity is frequently found in a small geographic area, diversity in open vegetation domains appear to be reduced to isolated patches, like a mosaic with different species compositions showing a variety of diversity levels, associated to speciation of some animal taxa during the Quaternary-Tertiary and affecting the distribution of *T. apereoides*, *T. inermis* and T. *laurentius* species complexes. This finding has implications for conservation, suggesting that a higher number of conservation units should be created in the Cerrado to account for the preservation of extant biodiversity.

## Supporting Information

Figure S1 Maximum likelihood phylogeny for *cytb* of *Thrichomys*. Similar topology was observed for Bayesian analysis. Numbers close to branches are SH-aLRT followed by posterior probability (pp) values. When identical values are observed, only one value is shown.
(PDF)

Figure S2 Maximum likelihood phylogeny for FGB of *Thrichomys*. Similar topology was observed for Bayesian analysis. Numbers close to branches are SH-aLRT followed by pp values. When identical values are observed, only one value is shown.
(PDF)

Figure S3 Summary of Bayes-DIVA analysis of *Thrichomys*. The tree is a MCC phylogeny generated with Beast for *cytb*. Circles at nodes show probabilities of ancestral ranges. Only the higher probabilities are shown. When two biogeographic regions are underlined in a node, it represents that ancestral range was at both regions. Biogeographic regions: A: southern Caatinga; B: northern Caatinga; C: central Caatinga; D: southern Cerrado; E: northern Cerrado; F: central Cerrado (see map in top left and Table S1).
(PDF)

Table S1 List of *Thrichomys* specimens included in this study, their haplotype number (H), GenBank accession number, field or museum identification number (ID), localities, karyotypes and Biogeographic regions (Bio Regions).
(PDF)
