# Supplementary material for: The Role of Historical Barriers in the Diversification Processes in Open Vegetation Formations during the Miocene/Pliocene Using an Ancient Rodent Lineage as a Model
Source: PLoS One. 2013 Apr 18;8(4):e61924. doi: 10.1371/journal.pone.0061924 (PMC3630152; doi:10.1371/journal.pone.0061924)
Supplement: Figure S1 — Maximum likelihood phylogeny for cytb of Thrichomys. Similar topology was observed for Bayesian analysis. Numbers close to branches are SH-aLRT followed by posterior probability (pp) values. When identical values are observed, only one value is shown. (PDF) [file pone.0061924.s001.pdf]

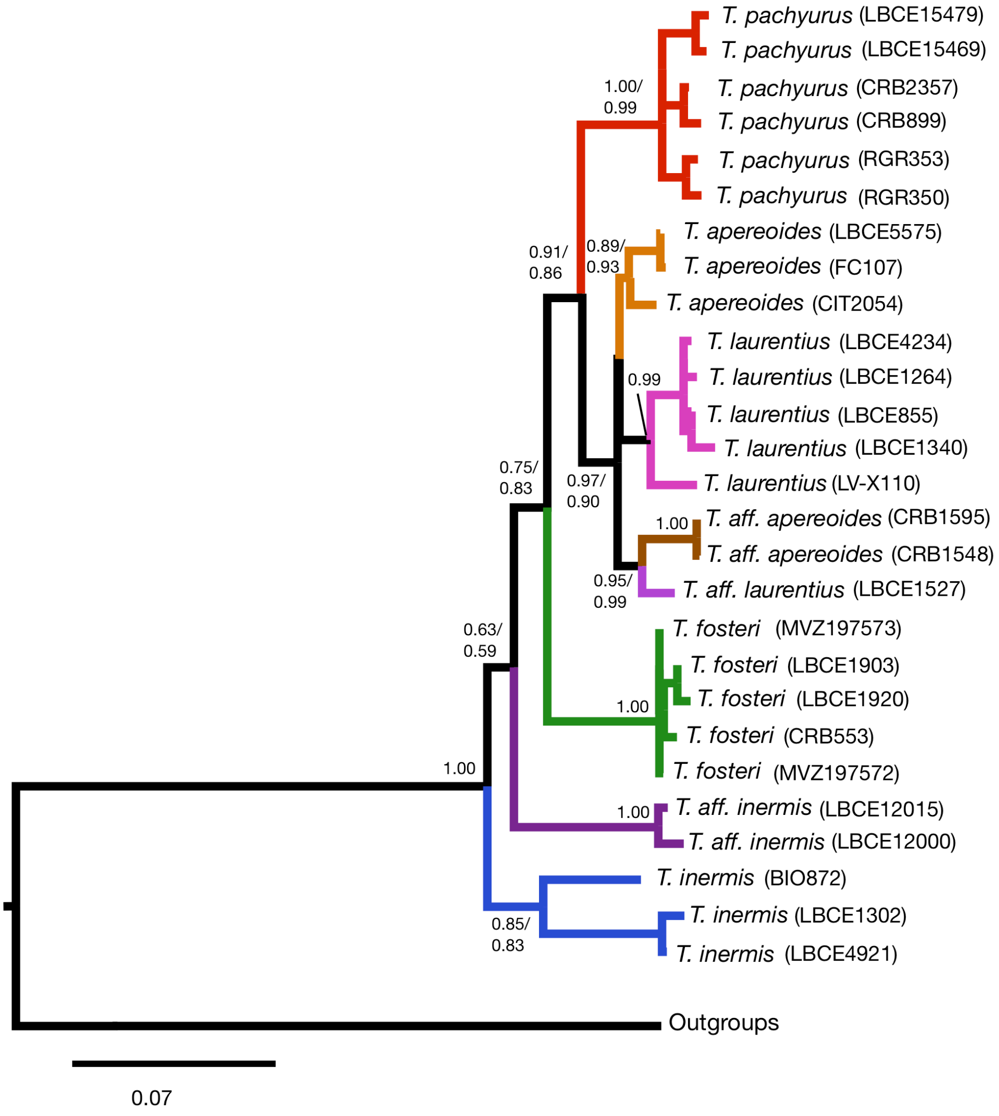

Figure S1 Maximum likelihood phylogeny for *cytb* of *Thrichomys*. Similar topology was observed for Bayesian analysis. Numbers close to branches are SH-aLRT followed by posterior probability (pp) values. When identical values are observed, only one value is shown.
