# Supplementary material for: The Role of Historical Barriers in the Diversification Processes in Open Vegetation Formations during the Miocene/Pliocene Using an Ancient Rodent Lineage as a Model
Source: PLoS One. 2013 Apr 18;8(4):e61924. doi: 10.1371/journal.pone.0061924 (PMC3630152; doi:10.1371/journal.pone.0061924)
Supplement: Figure S3 — Summary of Bayes-DIVA analysis of Thrichomys. The tree is a MCC phylogeny generated with Beast for cytb. Circles at nodes show probabilities of ancestral ranges. Only the higher probabilities are shown. When two biogeographic regions are underlined in a node, it represents that ancestral range was at both regions. Biogeographic regions: A: southern Caatinga; B: northern Caatinga; C: central Caatinga; D: southern Cerrado; E: northern Cerrado; F: central Cerrado (see map in top left and Table S1). (PDF) [file pone.0061924.s003.pdf]

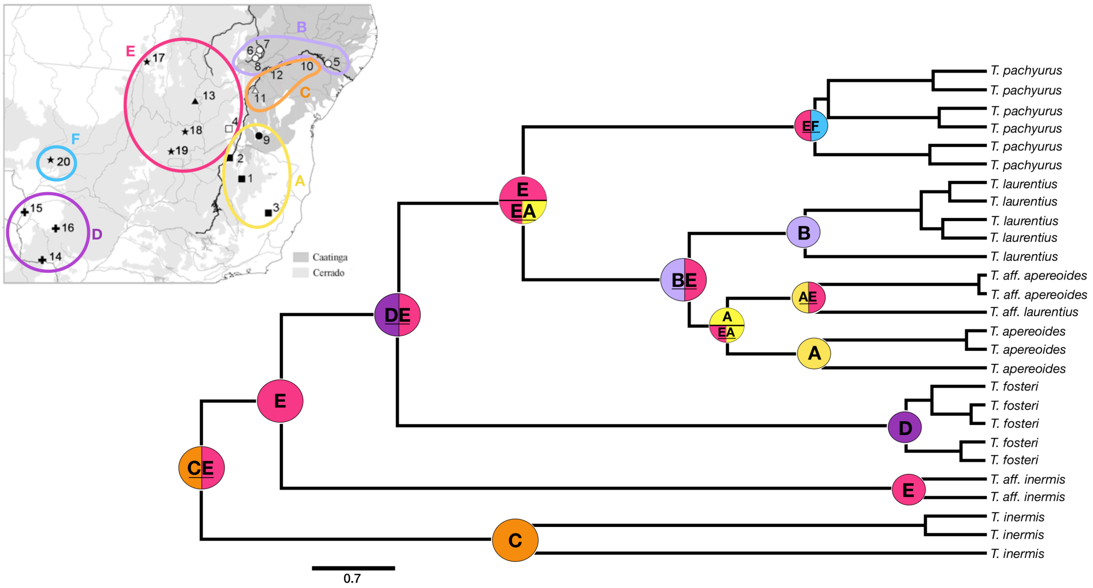

Figure S3 Summary of Bayes-DIVA analysis of *Thrichomys*. The tree is a MCC phylogeny generated with BEAST for *cytb*. Circles at nodes show probabilities of ancestral ranges. Only the higher probabilities are shown. When two biogeographic regions are underlined in a node, it represents that ancestral range was at both regions. Biogeographic regions: A: southern Caatinga; B: northern Caatinga; C: central Caatinga; D: southern Cerrado; E: northern Cerrado; F: central Cerrado (see map in top left and Table S1).
