# Supplementary material for: The Role of Historical Barriers in the Diversification Processes in Open Vegetation Formations during the Miocene/Pliocene Using an Ancient Rodent Lineage as a Model
Source: PLoS One. 2013 Apr 18;8(4):e61924. doi: 10.1371/journal.pone.0061924 (PMC3630152; doi:10.1371/journal.pone.0061924)
Supplement: Table S1 — List of Thrichomys specimens included in this study, their haplotype number (H), GenBank accession number, field or museum identification number (ID), localities, karyotypes and Biogeographic regions (Bio Regions). (PDF) [file pone.0061924.s004.pdf]

Table S1 List of *Thrichomys* specimens included in this study, their haplotype number (H), GenBank accession number, field or museum identification number (ID), localities, karyotypes and Biogeographic regions (Bio Regions).

			cytb		FBG				
**Species**	**ID**	**H**	**bp**	**GenBank**	**bp**	**GenBank**	**Locality (number in map)**	**2n/FN**	**Bio Regions**
T. apereoides	FC107	1	1100	AY083341			MG: Juramento (1)	28/50	Southern Caatinga
T. apereoides	CIT2054	2	1140	EU544668			MG: Januária (2)	?	Southern Caatinga
T. apereoides	LBCE5560	3	1140	JX459852	743	JX459844	MG: Capitão Andrade (3)	-	Southern Caatinga
T. apereoides	LBCE5561	3	1140	JX459853	744	JX459831	MG: Capitão Andrade (3)	28/50	Southern Caatinga
T. apereoides	LBCE5575	3	1140	JX459854	743	JX459843	MG: Capitão Andrade (3)	-	Southern Caatinga
T. aff. apereoides	CRB1548	4	1085	AY083337			BA: Jaborandi (4)	-	Northern Cerrado
T. aff. apereoides	CRB1559	5	1110	AY083336			BA: Jaborandi (4)	-	Northern Cerrado
T. aff. apereoides	CRB1595	5	1110	AY083342	743	JX459848	BA: Jaborandi (4)	-	Northern Cerrado
T. aff. apereoides	CRB1848	5	993	AY083339			BA: Jaborandi (4)	-	Northern Cerrado
T. aff. apereoides	CRB1876	6	1031	JX459855			BA: Jaborandi (4)	28/52	Northern Cerrado
T. laurentius	LV-X102	7	1140	AY083338			AL: Delmiro Gouveia (5)	?	Northern Caatinga
T. laurentius	LBCE852	8	1140	JX459856			PI: Coronel José Dias (6)	-	Northern Caatinga
T. laurentius	LBCE854	9	1140	JX459857			PI: Coronel José Dias (6)	-	Northern Caatinga
T. laurentius	LBCE855	10	1140	JX459858	744	JX459834	PI: Coronel José Dias (6)	-	Northern Caatinga
T. laurentius	LBCE856	11	1140	AY083333			PI: Coronel José Dias (6)	30/54	Northern Caatinga
T. laurentius	LBCE857	12	1140	JX459859			PI: Coronel José Dias (6)	-	Northern Caatinga
T. laurentius	LBCE859	13	1140	JX459860			PI: Coronel José Dias (6)	-	Northern Caatinga
T. laurentius	LBCE861	14	1140	JX459861			PI: Coronel José Dias (6)	-	Northern Caatinga
T. laurentius	LBCE862	15	1140	AY083334			PI: Coronel José Dias (6)	-	Northern Caatinga
T. laurentius	LBCE880	16	1140	JX459862			PI: Coronel José Dias (6)	-	Northern Caatinga
T. laurentius	LBCE889	17	1140	JX459863			PI: Coronel José Dias (6)	-	Northern Caatinga
T. laurentius	LBCE890	18	1140	JX459864			PI: Coronel José Dias (6)	-	Northern Caatinga

T. laurentius	LBCE1340	19	700	AY083335	744	JX459836	PI: Coronel José Dias (6)	-	Northern Caatinga
T. laurentius	LBCE1231	20	1140	JX459865			PI: João Costa (7)	?	Northern Caatinga
T. laurentius	LBCE1232	21	1140	JX459866			PI: João Costa (7)	?	Northern Caatinga
T. laurentius	LBCE1234	21	1140	JX459867			PI: João Costa (7)	?	Northern Caatinga
T. laurentius	LBCE1241	22	1140	JX459868			PI: João Costa (7)	?	Northern Caatinga
T. laurentius	LBCE1243	23	1140	JX459869			PI: João Costa (7)	?	Northern Caatinga
T. laurentius	LBCE1244	24	1140	JX459870			PI: João Costa (7)	?	Northern Caatinga
T. laurentius	LBCE1246	25	1140	JX459871			PI: João Costa (7)	?	Northern Caatinga
T. laurentius	LBCE1248	26	1140	JX459872			PI: João Costa (7)	?	Northern Caatinga
T. laurentius	LBCE1256	27	1140	JX459873			PI: João Costa (7)	?	Northern Caatinga
T. laurentius	LBCE1258	28	1140	JX459874			PI: João Costa (7)	?	Northern Caatinga
T. laurentius	LBCE1262	29	1140	JX459875			PI: João Costa (7)	?	Northern Caatinga
T. laurentius	LBCE1264	30	1140	JX459876			PI: João Costa (7)	?	Northern Caatinga
T. laurentius	LBCE1257	31	1140	AY083332			PI: João Costa (7)	?	Northern Caatinga
T. laurentius	LBCE4231	31	1140	JX459878			PI: São Raimundo Nonato (8)	-	Northern Caatinga
T. laurentius	LBCE4241	31	1140	JX459881			PI: São Raimundo Nonato (8)	-	Northern Caatinga
T. laurentius	LBCE4230	32	1140	JX459877			PI: São Raimundo Nonato (8)	-	Northern Caatinga
T. laurentius	LBCE4234	33	1140	JX459879	744	JX459835	PI: São Raimundo Nonato (8)	-	Northern Caatinga
T. laurentius	LBCE4238	34	1140	JX459880			PI: São Raimundo Nonato (8)	-	Northern Caatinga
T. laurentius	LBCE4245	35	1140	JX459882			PI: São Raimundo Nonato (8)	-	Northern Caatinga
T. laurentius	LBCE4253	36	1140	JX459883			PI: São Raimundo Nonato (8)	-	Northern Caatinga
T. laurentius	LBCE4262	37	1140	JX459884			PI: São Raimundo Nonato (8)	-	Northern Caatinga
T. aff. laurentius	LBCE1505	38	1140	AY083344	743	JX459832	BA: Caetité (9)	30/54	Southern Caatinga
T. aff. laurentius	LBCE1527	38	1140	JX459885			BA: Caetité (9)	-	Southern Caatinga
T. inermis	LBCE4906	39	1140	JX459886			BA: Curaçá (10)	-	Central Caatinga
T. inermis	LBCE4921	40	1140	JX459887	743	JX459845	BA: Curaçá (10)	-	Central Caatinga
T. inermis	LBCE1302	41	802	AY083343	744	JX459847	BA: Sento Sé (11)	26/48	Central Caatinga
T. inermis	BIO 872	42	1140	U34855			BA: Santo Inácio (12)	?	Central Caatinga

T. aff. inermis	LBCE12000	43	1140	JX459888	744	JX459841	TO: Novo Jardim (13)	26/48	Northern Cerrado
T. aff. inermis	LBCE12001	43	1140	JX459889	744	JX459837	TO: Novo Jardim (13)	26/48	Northern Cerrado
T. aff. inermis	LBCE12020	43	1140	JX459893	744	JX459842	TO: Novo Jardim (13)	26/48	Northern Cerrado
T. aff. inermis	LBCE12015	44	1140	JX459891	744	JX459838	TO: Novo Jardim (13)	26/48	Northern Cerrado
T. aff. inermis	LBCE12009	45	1140	JX459890			TO: Novo Jardim (13)	26/48	Northern Cerrado
T. aff. inermis	LBCE12017	46	1140	JX459892			TO: Novo Jardim (13)	26/48	Northern Cerrado
T. fosteri	CRB553	47	1140	JX459894			MS: Bela Vista (14)	?	Southern Cerrado
T. fosteri	LBCE1920	48	1140	AY083329	744	JX459833	MS: Corumbá (15)	34/64	Southern Cerrado
T. fosteri	LBCE1903	49	1096	AY083328	744	JX459846	MS: Corumbá (15)	-	Southern Cerrado
T. fosteri	LBCE1960	49	1123	AY083340			MS: Corumbá (15)	34/64	Southern Cerrado
T. fosteri	MVZ197573	50	798	EU313253			MS: Fazenda Taboco (16)	?	Southern Cerrado
T. fosteri	MVZ197572	51	798	EU313252			MS: Fazenda Taboco (16)	?	Southern Cerrado
T. pachyurus	RGR350	52	801	HM594625			TO: Caseara (17)	?	Northern Cerrado
T. pachyurus	RGR353	53	797	HM594626			TO: Caseara (17)	?	Northern Cerrado
T. pachyurus	CRB899	54	1109	AY083331			GO: Teresina de Goiás (18)	30/56	Northern Cerrado
T. pachyurus	CRB2357	55	1140	JX459895			GO: Mimoso de Goiás (19)	?	Northern Cerrado
T. pachyurus	LBCE15469	56	1140	JX459896			MT: Cuiaba (20)	30/56	Central Cerrado
T. pachyurus	LBCE15471	57	1140	JX459897	743	JX459839	MT: Cuiaba (20)	30/56	Central Cerrado
T. pachyurus	LBCE15477	58	1140	JX459898			MT: Cuiaba (20)	30/56	Central Cerrado
T. pachyurus	LBCE15479	59	1140	JX459899	743	JX459840	MT: Cuiaba (20)	30/56	Central Cerrado

Brazilian states corresponds to MG = Minas Gerais, BA = Bahia, AL = Alagoas, PI = Piauí, TO = Tocantins, MS = Mato Grosso do Sul, GO = Goiás, MT = Mato Grosso; - = karyotype known for the locality, but not available for these specimens, ? = karyotype from the locality is unknown
